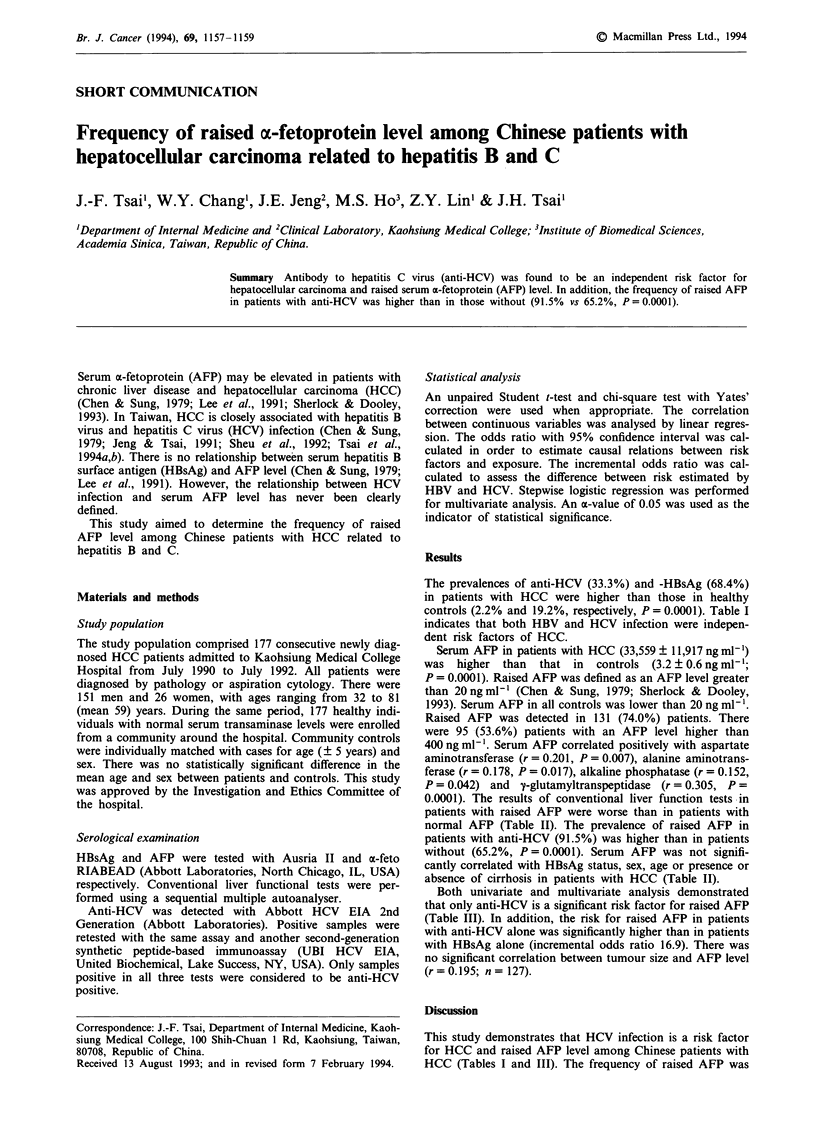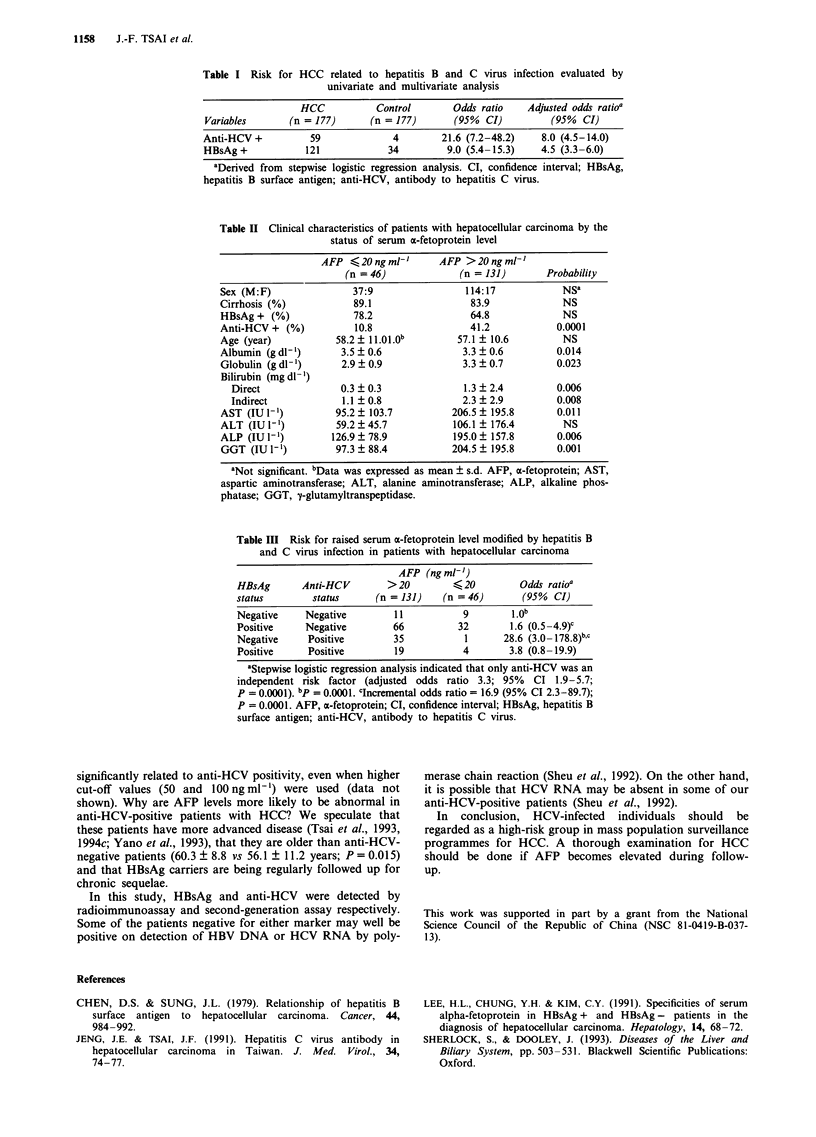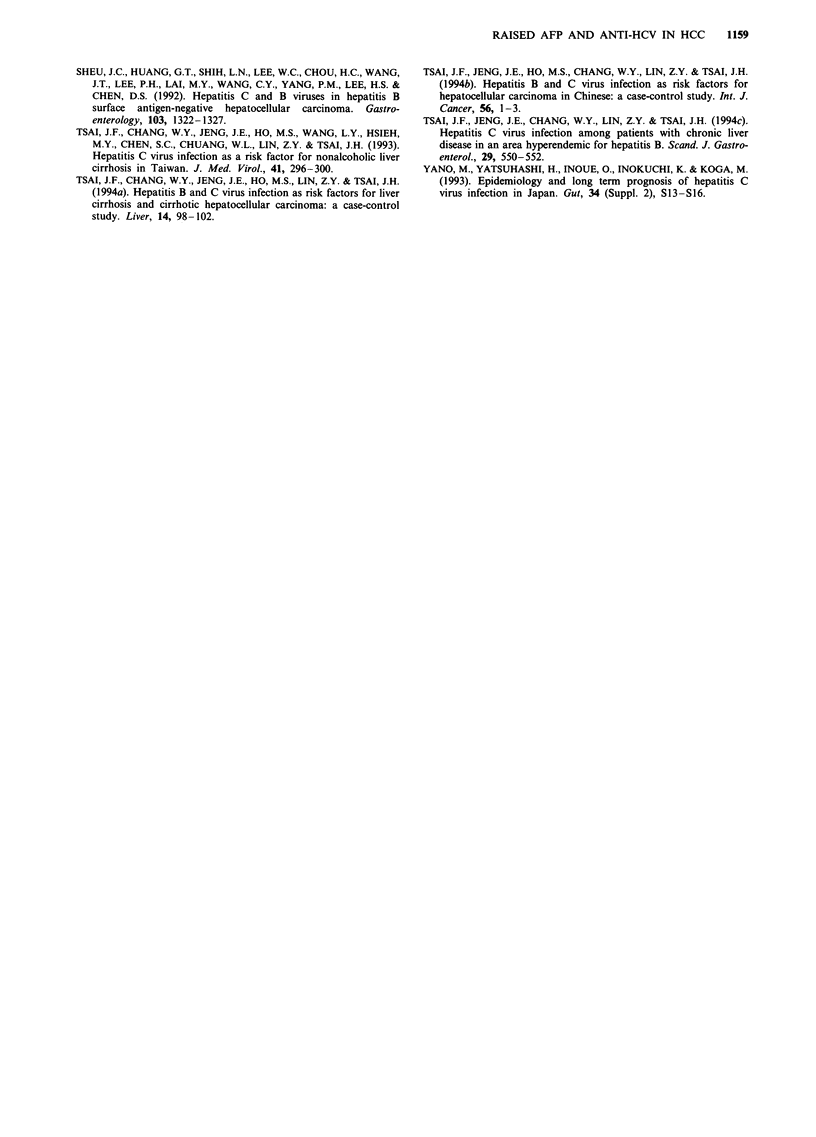# Frequency of raised alpha-fetoprotein level among Chinese patients with hepatocellular carcinoma related to hepatitis B and C.

**DOI:** 10.1038/bjc.1994.227

**Published:** 1994-06

**Authors:** J. F. Tsai, W. Y. Chang, J. E. Jeng, M. S. Ho, Z. Y. Lin, J. H. Tsai

**Affiliations:** Department of Internal Medicine, Kaohsiung Medical College, Taiwan, Republic of China.

## Abstract

Antibody to hepatitis C virus (anti-HCV) was found to be an independent risk factor for hepatocellular carcinoma and raised serum alpha-fetoprotein (AFP) level. In addition, the frequency of raised AFP in patients with anti-HCV was higher than in those without (91.5% vs 65.2%, P = 0.0001).


Br. J. Cancer (1994), 69, 1157-1159 © Macmillan Press Ltd., 1994

SHORT COMMUNICATION

### Frequency of raised α-fetoprotein level among Chinese patients with hepatocellular carcinoma related to hepatitis B and C

J.-F. Tsai[1], W.Y. Chang[1], J.E. Jeng[2], M.S. Ho[3], Z.Y. Lin[1] & J.H. Tsai[1]

[1]*Department of Internal Medicine and* [2]*Clinical Laboratory, Kaohsiung Medical College;* [3]*Institute of Biomedical Sciences, Academia Sinica, Taiwan, Republic of China.*

**Summary** Antibody to hepatitis C virus (anti-HCV) was found to be an independent risk factor for hepatocellular carcinoma and raised serum α-fetoprotein (AFP) level. In addition, the frequency of raised AFP in patients with anti-HCV was higher than in those without (91.5% *vs* 65.2%, $P = 0.0001$).

Serum α-fetoprotein (AFP) may be elevated in patients with chronic liver disease and hepatocellular carcinoma (HCC) (Chen & Sung, 1979; Lee *et al.*, 1991; Sherlock & Dooley, 1993). In Taiwan, HCC is closely associated with hepatitis B virus and hepatitis C virus (HCV) infection (Chen & Sung, 1979; Jeng & Tsai, 1991; Sheu *et al.*, 1992; Tsai *et al.*, 1994*a,b*). There is no relationship between serum hepatitis B surface antigen (HBsAg) and AFP level (Chen & Sung, 1979; Lee *et al.*, 1991). However, the relationship between HCV infection and serum AFP level has never been clearly defined.

This study aimed to determine the frequency of raised AFP level among Chinese patients with HCC related to hepatitis B and C.

### Materials and methods

### *Study population*

The study population comprised 177 consecutive newly diagnosed HCC patients admitted to Kaohsiung Medical College Hospital from July 1990 to July 1992. All patients were diagnosed by pathology or aspiration cytology. There were 151 men and 26 women, with ages ranging from 32 to 81 (mean 59) years. During the same period, 177 healthy individuals with normal serum transaminase levels were enrolled from a community around the hospital. Community controls were individually matched with cases for age (± 5 years) and sex. There was no statistically significant difference in the mean age and sex between patients and controls. This study was approved by the Investigation and Ethics Committee of the hospital.

### *Serological examination*

HBsAg and AFP were tested with Ausria II and α-feto RIABEAD (Abbott Laboratories, North Chicago, IL, USA) respectively. Conventional liver functional tests were performed using a sequential multiple autoanalyser.

Anti-HCV was detected with Abbott HCV EIA 2nd Generation (Abbott Laboratories). Positive samples were retested with the same assay and another second-generation synthetic peptide-based immunoassay (UBI HCV EIA, United Biochemical, Lake Success, NY, USA). Only samples positive in all three tests were considered to be anti-HCV positive.

Correspondence: J.-F. Tsai, Department of Internal Medicine, Kaohsiung Medical College, 100 Shih-Chuan 1 Rd, Kaohsiung, Taiwan, 80708, Republic of China.
Received 13 August 1993; and in revised form 7 February 1994.

### *Statistical analysis*

An unpaired Student *t*-test and chi-square test with Yates' correction were used when appropriate. The correlation between continuous variables was analysed by linear regression. The odds ratio with 95% confidence interval was calculated in order to estimate causal relations between risk factors and exposure. The incremental odds ratio was calculated to assess the difference between risk estimated by HBV and HCV. Stepwise logistic regression was performed for multivariate analysis. An α-value of 0.05 was used as the indicator of statistical significance.

### Results

The prevalences of anti-HCV (33.3%) and -HBsAg (68.4%) in patients with HCC were higher than those in healthy controls (2.2% and 19.2%, respectively, $P = 0.0001$). Table I indicates that both HBV and HCV infection were independent risk factors of HCC.

Serum AFP in patients with HCC ($33,559 \pm 11,917$ ng ml$^{-1}$) was higher than that in controls ($3.2 \pm 0.6$ ng ml$^{-1}$; $P = 0.0001$). Raised AFP was defined as an AFP level greater than 20 ng ml$^{-1}$ (Chen & Sung, 1979; Sherlock & Dooley, 1993). Serum AFP in all controls was lower than 20 ng ml$^{-1}$. Raised AFP was detected in 131 (74.0%) patients. There were 95 (53.6%) patients with an AFP level higher than 400 ng ml$^{-1}$. Serum AFP correlated positively with aspartate aminotransferase ($r = 0.201$, $P = 0.007$), alanine aminotransferase ($r = 0.178$, $P = 0.017$), alkaline phosphatase ($r = 0.152$, $P = 0.042$) and γ-glutamyltranspeptidase ($r = 0.305$, $P = 0.0001$). The results of conventional liver function tests in patients with raised AFP were worse than in patients with normal AFP (Table II). The prevalence of raised AFP in patients with anti-HCV (91.5%) was higher than in patients without (65.2%, $P = 0.0001$). Serum AFP was not significantly correlated with HBsAg status, sex, age or presence or absence of cirrhosis in patients with HCC (Table II).

Both univariate and multivariate analysis demonstrated that only anti-HCV is a significant risk factor for raised AFP (Table III). In addition, the risk for raised AFP in patients with anti-HCV alone was significantly higher than in patients with HBsAg alone (incremental odds ratio 16.9). There was no significant correlation between tumour size and AFP level ($r = 0.195$; $n = 127$).

### Discussion

This study demonstrates that HCV infection is a risk factor for HCC and raised AFP level among Chinese patients with HCC (Tables I and III). The frequency of raised AFP was

**Table I** Risk for HCC related to hepatitis B and C virus infection evaluated by univariate and multivariate analysis

| Variables | HCC (n = 177) | Control (n = 177) | Odds ratio (95% CI) | Adjusted odds ratio[a] (95% CI) |
|---|---|---|---|---|
| Anti-HCV + | 59 | 4 | 21.6 (7.2–48.2) | 8.0 (4.5–14.0) |
| HBsAg + | 121 | 34 | 9.0 (5.4–15.3) | 4.5 (3.3–6.0) |

[a]Derived from stepwise logistic regression analysis. CI, confidence interval; HBsAg, hepatitis B surface antigen; anti-HCV, antibody to hepatitis C virus.

**Table II** Clinical characteristics of patients with hepatocellular carcinoma by the status of serum α-fetoprotein level

| | AFP $\leqslant 20\ ng\ ml^{-1}$ (n = 46) | AFP $> 20\ ng\ ml^{-1}$ (n = 131) | Probability |
|---|---|---|---|
| Sex (M:F) | 37:9 | 114:17 | NS[a] |
| Cirrhosis (%) | 89.1 | 83.9 | NS |
| HBsAg + (%) | 78.2 | 64.8 | NS |
| Anti-HCV + (%) | 10.8 | 41.2 | 0.0001 |
| Age (year) | 58.2 ± 11.01.0[b] | 57.1 ± 10.6 | NS |
| Albumin ($g\ dl^{-1}$) | 3.5 ± 0.6 | 3.3 ± 0.6 | 0.014 |
| Globulin ($g\ dl^{-1}$) | 2.9 ± 0.9 | 3.3 ± 0.7 | 0.023 |
| Bilirubin ($mg\ dl^{-1}$) | | | |
| Direct | 0.3 ± 0.3 | 1.3 ± 2.4 | 0.006 |
| Indirect | 1.1 ± 0.8 | 2.3 ± 2.9 | 0.008 |
| AST ($IU\ l^{-1}$) | 95.2 ± 103.7 | 206.5 ± 195.8 | 0.011 |
| ALT ($IU\ l^{-1}$) | 59.2 ± 45.7 | 106.1 ± 176.4 | NS |
| ALP ($IU\ l^{-1}$) | 126.9 ± 78.9 | 195.0 ± 157.8 | 0.006 |
| GGT ($IU\ l^{-1}$) | 97.3 ± 88.4 | 204.5 ± 195.8 | 0.001 |

[a]Not significant. [b]Data was expressed as mean ± s.d. AFP, α-fetoprotein; AST, aspartic aminotransferase; ALT, alanine aminotransferase; ALP, alkaline phosphatase; GGT, γ-glutamyltranspeptidase.

**Table III** Risk for raised serum α-fetoprotein level modified by hepatitis B and C virus infection in patients with hepatocellular carcinoma

| HBsAg status | Anti-HCV status | AFP ($ng\ ml^{-1}$) $>20$ (n = 131) | AFP ($ng\ ml^{-1}$) $\leqslant 20$ (n = 46) | Odds ratio[a] (95% CI) |
|---|---|---|---|---|
| Negative | Negative | 11 | 9 | 1.0[b] |
| Positive | Negative | 66 | 32 | 1.6 (0.5–4.9)[c] |
| Negative | Positive | 35 | 1 | 28.6 (3.0–178.8)[b,c] |
| Positive | Positive | 19 | 4 | 3.8 (0.8–19.9) |

[a]Stepwise logistic regression analysis indicated that only anti-HCV was an independent risk factor (adjusted odds ratio 3.3; 95% CI 1.9–5.7; $P = 0.0001$). [b]$P = 0.0001$. [c]Incremental odds ratio = 16.9 (95% CI 2.3–89.7); $P = 0.0001$. AFP, α-fetoprotein; CI, confidence interval; HBsAg, hepatitis B surface antigen; anti-HCV, antibody to hepatitis C virus.

significantly related to anti-HCV positivity, even when higher cut-off values (50 and 100 $ng\ ml^{-1}$) were used (data not shown). Why are AFP levels more likely to be abnormal in anti-HCV-positive patients with HCC? We speculate that these patients have more advanced disease (Tsai *et al.*, 1993, 1994*c*; Yano *et al.*, 1993), that they are older than anti-HCV-negative patients (60.3 ± 8.8 *vs* 56.1 ± 11.2 years; $P = 0.015$) and that HBsAg carriers are being regularly followed up for chronic sequelae.

In this study, HBsAg and anti-HCV were detected by radioimmunoassay and second-generation assay respectively. Some of the patients negative for either marker may well be positive on detection of HBV DNA or HCV RNA by polymerase chain reaction (Sheu *et al.*, 1992). On the other hand, it is possible that HCV RNA may be absent in some of our anti-HCV-positive patients (Sheu *et al.*, 1992).

In conclusion, HCV-infected individuals should be regarded as a high-risk group in mass population surveillance programmes for HCC. A thorough examination for HCC should be done if AFP becomes elevated during follow-up.

This work was supported in part by a grant from the National Science Council of the Republic of China (NSC 81-0419-B-037-13).

### References

CHEN, D.S. & SUNG, J.L. (1979). Relationship of hepatitis B surface antigen to hepatocellular carcinoma. *Cancer*, **44**, 984–992.

JENG, J.E. & TSAI, J.F. (1991). Hepatitis C virus antibody in hepatocellular carcinoma in Taiwan. *J. Med. Virol.*, **34**, 74–77.

LEE, H.L., CHUNG, Y.H. & KIM, C.Y. (1991). Specificities of serum alpha-fetoprotein in HBsAg + and HBsAg – patients in the diagnosis of hepatocellular carcinoma. *Hepatology*, **14**, 68–72.

SHERLOCK, S., & DOOLEY, J. (1993). *Diseases of the Liver and Biliary System*, pp. 503–531. Blackwell Scientific Publications: Oxford.

SHEU, J.C., HUANG, G.T., SHIH, L.N., LEE, W.C., CHOU, H.C., WANG, J.T., LEE, P.H., LAI, M.Y., WANG, C.Y., YANG, P.M., LEE, H.S. & CHEN, D.S. (1992). Hepatitis C and B viruses in hepatitis B surface antigen-negative hepatocellular carcinoma. *Gastroenterology*, **103,** 1322–1327.

TSAI, J.F., CHANG, W.Y., JENG, J.E., HO, M.S., WANG, L.Y., HSIEH, M.Y., CHEN, S.C., CHUANG, W.L., LIN, Z.Y. & TSAI, J.H. (1993). Hepatitis C virus infection as a risk factor for nonalcoholic liver cirrhosis in Taiwan. *J. Med. Virol.*, **41,** 296–300.

TSAI, J.F., CHANG, W.Y., JENG, J.E., HO, M.S., LIN, Z.Y. & TSAI, J.H. (1994*a*). Hepatitis B and C virus infection as risk factors for liver cirrhosis and cirrhotic hepatocellular carcinoma: a case-control study. *Liver*, **14,** 98–102.

TSAI, J.F., JENG, J.E., HO, M.S., CHANG, W.Y., LIN, Z.Y. & TSAI, J.H. (1994*b*). Hepatitis B and C virus infection as risk factors for hepatocellular carcinoma in Chinese: a case-control study. *Int. J. Cancer*, **56,** 1–3.

TSAI, J.F., JENG, J.E., CHANG, W.Y., LIN, Z.Y. & TSAI, J.H. (1994*c*). Hepatitis C virus infection among patients with chronic liver disease in an area hyperendemic for hepatitis B. *Scand. J. Gastroenterol.*, **29,** 550–552.

YANO, M., YATSUHASHI, H., INOUE, O., INOKUCHI, K. & KOGA, M. (1993). Epidemiology and long term prognosis of hepatitis C virus infection in Japan. *Gut*, **34** (Suppl. 2), S13–S16.